# Supplementary material for: Lung Ultrasound Improves Outcome Prediction over Clinical Judgment in COVID-19 Patients Evaluated in the Emergency Department
Source: J Clin Med. 2022 May 27;11(11):3032. doi: 10.3390/jcm11113032 (PMC9181775; doi:10.3390/jcm11113032)
Supplement: Supplementary file 1 [file jcm-11-03032-s001.zip › jcm-1681803-supplementary.pdf]

Lung Ultrasound Improves Outcome Prediction over Clinical Judgment in COVID-19 Patients Evaluated in the Emergency Department

Supplementary Materials

CODED Study Investigators not included in the author list:

Arianna Ardito [1], Gilberto Calzolari [1], Dario Leone [1], Stefania Locatelli [1], Monica Masoero [1], Maria Tizzani [1], Andrea Viola [1], Margherita Calicchia [1,2], Ilenia Carretto [1,2], Maria Gelardi [1,2], Eleonora Dipietro [1,2], Matteo Oddi [1,2], Virginia Scategni [1,2], Astrid Cecilia Surra [1,2], Gabriele Cerini [3], Carolina Pelliccioni [3] and Alessandra Ricciardolo [3].

Affiliations:

1. S.C. Medicina d'Urgenza U, Ospedale Molinette, A.O.U. Città della Salute e della Scienza di Torino, 10126 Torino, Italy
2. Scuola di Specializzazione in Medicina d'Emergenza-Urgenza, Università degli Studi di Torino, 10126 Torino, Italy
3. Medicina e Chirurgia di Urgenza e Accettazione, A.O.U. Careggi, 50134 Firenze, Italy

Supplementary Table S1. Variables in the HOME-CoV rule, adapted from Duillet D. et al. [7]. Presence of one or more criteria corresponds to a POSITIVE HOME-CoV rule.

**Clinical Variable**
Pulse oxygen saturation ≤94% in ambient air
Respiratory rate ≥25/min
Ability to talk without breathing <8 sec.
Systolic blood pressure ≤90 mmHg
Heart rate ≥120 beats/min
Confusion or impaired consciousness
Severe comorbidity[a] and inadequate living conditions[b]

A: Severe chronic respiratory disease (unstable asthma, COPD stage III or IV, respiratory failure with continuous oxygen therapy), chronic heart failure (New York Heart Association class ≥III), severe cognitive disorder, or immunodepression (primary immunodeficiency, uncontrolled HIV infection, immunosuppressive drug, chemotherapy). B: Inappropriate dwelling (homeless, frail relative at home, long-term care institution), lack of support person (family member or friend), or home follow-up impossible.

Supplementary Table S2. 4C Mortality Score composition, adapted from Knight S.R. et al. [8].

**Variable**	**N. points**
Age, years	
<50	0
50-59	2
60-69	4
70-79	6
≥80	7
Sex	
Female	0
Male	1
N. of comorbidities*	
0	0
1	1
≥2	2
Respiratory rate, breaths/min	
<20	0
20-29	1
≥30	2
Peripheral oxygen saturation on room air	
≥92%	0
<92%	2
Glasgow Coma Scale	
=15	0
<15	2
Urea, mmol/L	
<7	0
7-14	1
>14	3
OR	
Creatinine^ mg/dL	
<0.97	0
0.97-1.69	1
>1.69	3
C-reactive protein, mg/L (mg/dL)	
<50 (5)	0
50-99 (5-9.9)	1
≥100 (10)	2

**per* Charlson comorbidity index: chronic cardiac disease, chronic respiratory disease, chronic renal disease (eGFR ≤30 ml/min), mild to severe liver disease, dementia, chronic neurological conditions, diabetes mellitus.

^For centers lacking urea assay.

Supplementary Table S3. Cross-tabulation of LUS data and 30-day clinical outcomes.

	**Primary composite outcome**	**Secondary composite outcome**	Subsequent ED visit	Hospital admission	Supplemental O_2	NIV	Death
All patients (n=393)	35 (8.9%)	14 (3.6%)	48 (12.2%)	35 (8.9%)	32 (8.1%)	10 (2.5%)	2 (0.5%)
LUS modified score							
**0** (n=234)	7 (3.0%)	2 (0.9%)	14 (6.0%)	7 (3.0%)	6 (2.6%)	2 (0.9%)	0 (0%)
**1** (n=40)	2 (5.0%)	1 (2.5%)	3 (7.5%)	2 (5.0%)	2 (5.0%)	1 (2.5%)	0 (0%)
**2** (n=62)	11 (17.7%)	2 (3.2%)	12 (19.4%)	11 (17.7%)	11 (17.7%)	2 (3.2%)	1 (1.6%)
**3** (n=15)	6 (40.0%)	3 (20.0%)	8 (53.3%)	6 (40.0%)	4 (26.7%)	1 (6.7%)	0 (0%)
**4** (n=27)	6 (22.2%)	5 (18.5%)	7 (25.9%)	6 (22.2%)	6 (22.2%)	3 (11.1%)	1 (3.7%)
**≥5** (n=15)	3 (20.0%)	1 (6.7%)	4 (26.7%)	3 (20.0%)	3 (20.0%)	1 (6.7%)	0 (0%)

% values refer to the number of patients in the corresponding risk-category.

Supplementary Table S4. Cross-tabulation of HOME-CoV/4CMS classification and 30-day clinical outcomes.

	**Primary composite outcome**	**Secondary composite outcome**	Subsequent ED visit	Hospital admission	Supplemental O_2	NIV	Death
All patients (n=393)	35 (8.9%)	14 (3.6%)	48 (12.2%)	35 (8.9%)	32 (8.1%)	10 (2.5%)	2 (0.5%)
HOME-CoV negative (n=273)	16 (5.9%)	6 (2.2%)	24 (8.8%)	16 (5.9%)	14 (5.1%)	4 (1.5%)	0 (0%)
HOME-CoV positive (n=120)	19 (15.8%)	8 (6.7%)	24 (20%)	19 (15.8%)	18 (15.0%)	6 (5.0%)	2 (1.7%)
4CMS 0-3 (low risk, n=243)	8 (3.3%)	2 (0.8%)	18 (7.4%)	8 (3.3%)	7 (2.9%)	2 (0.8%)	0 (0%)
4CMS 4-8 (intermediate risk, n=120)	19 (15.8%)	10 (8.3%)	22 (18.3%)	19 (15.8%)	19 (15.8%)	7 (5.8%)	1 (0.8%)
4CMS ≥9 (high risk, n=30)	8 (26.7%)	2 (6.7%)	8 (26.7%)	8 (26.7%)	6 (20.0%)	1 (3.3%)	1 (3.3%)

% values refer to the number of patients in the corresponding risk-category.

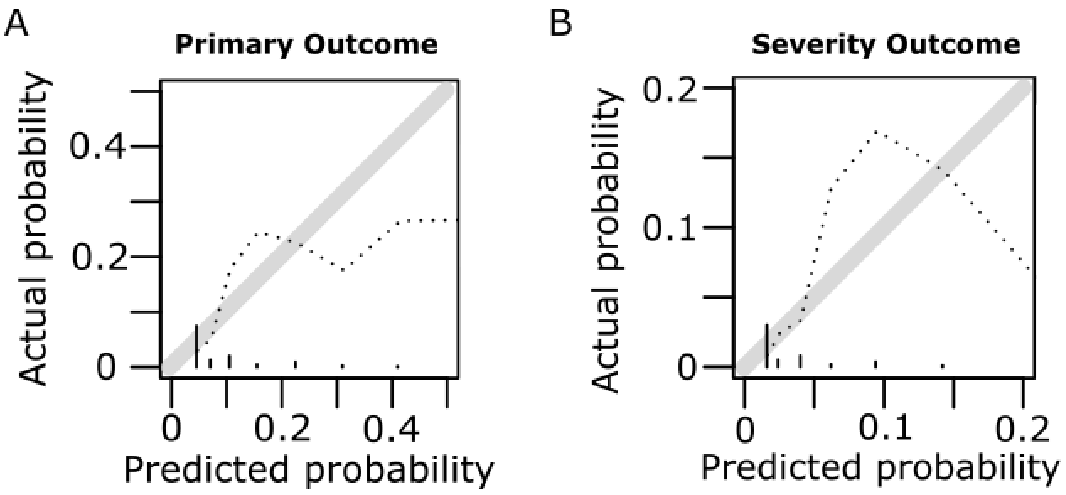

Supplementary Figure S1. Calibration plots of LUS for (**A**) the primary outcome and (**B**) secondary outcome.

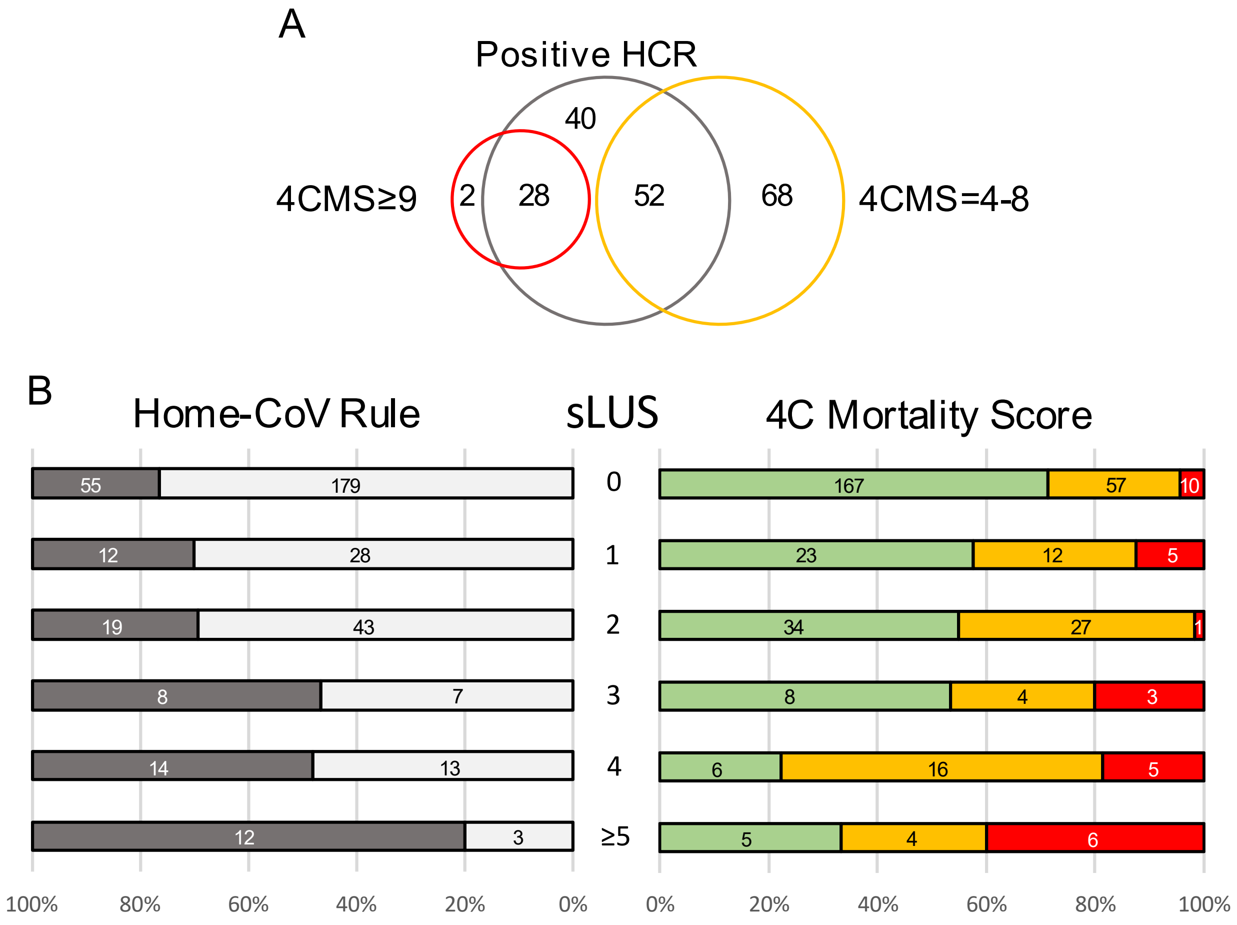

Supplementary Figure S2. Overlap of HCR, 4CMS and LUS results in study patients. **(A)** Venn's diagram. **(B)** Clinical and LUS classification. Green: low-risk (4CMS 0-3); yellow: intermediate-risk (4CMS 4-8); red: high-risk (4CMS ≥9).
